# Molecular cloning and expression of an encoding galactinol synthase gene (*AnGolS1*) in seedling of *Ammopiptanthus nanus*

**DOI:** 10.1038/srep36113

**Published:** 2016-10-27

**Authors:** YuDong Liu, Li Zhang, LiJing Chen, Hui Ma, YanYe Ruan, Tao Xu, ChuanQiang Xu, Yi He, MingFang Qi

**Affiliations:** 1 Horticulture Department, Shenyang Agricultural University, No. 120 Dongling Road, Shenhe District 110866, P.R. China; 2 Key Laboratory of Protected Horticulture of Ministry of Education, No. 120 Dongling Road, Shenhe District 110866, P.R. China; 3 Collaborative Innovation Center of Protected Vegetable Suround Bohai Gulf Region, No. 120 Dongling Road, Shenhe District 110866, P.R. China; 4 Key Laboratory of Agricultural Biotechnology of Liaoning Province, No. 120 Dongling Road, Shenhe District 110866, P.R. China; 5 Liaoning Plant Gene Engineering Research Center, No. 120 Dongling Road, Shenhe District 110866, P.R. China

## Abstract

Based on the galactinol synthase (*AnGolS1*) fragment sequence from a cold-induced Suppression Subtractive Hybridization (SSH) library derived from *Ammopiptanthus nanus (A. nanus*) seedlings, *AnGolS1* mRNA (including the 5′ UTR and 3′ UTR) (GenBank accession number: GU942748) was isolated and characterized by rapid amplification of cDNA ends polymerase chain reaction (RACE–PCR). A substrate reaction test revealed that AnGolS1 possessed galactinol synthase activity *in vitro* and could potentially be an early-responsive gene. Furthermore, quantitative real-time PCR (qRT-PCR) indicated that *AnGolS1* was responded to cold, salts and drought stresses, however, significantly up-regulated in all origans by low temperatures, especially in plant stems. In addition, the hybridization signals in the fascicular cambium were strongest in all cells under low temperature. Thus, we propose that *AnGolS1* plays critical roles in *A. nanus* low-temperature stress resistance and that fascicular cambium cells could be involved in *AnGolS1* mRNA transcription, galactinol transportation and coordination under low-temperature stress.

Environmental stresses are the most influence factors affecting plant survival and distribution worldwide^1^. Abiotic stresses, such as low temperature, drought, and salt stresses, can seriously affect the normal growth, development, and yield of plants^2^,^3^,^4^,^5^. To reduce the potential damage from these stresses, plants have evolved complex strategies, such as the accumulation of certain small soluble molecules (such as proline, mannitol, and oligosaccharide) to increase the osmotic pressure in cells, thereby improving plant stress resistance^6^,^7^. Raffinose family oligosaccharides (RFOs) are the second largest group of sugars in plant tissues and are nonstructural carbohydrates that play essential roles in plant growth and development^8^. In addition to enhancing abiotic stress tolerance, RFOs are an alternative to sucrose and starch for glucide transport and storage^9^,^10^.

In the galactinol-dependent pathway, RFOs are synthesized from sucrose and galactose precursors that are joined by an (α-1, 6) glycosidic bond. However, in the galactinol-independent pathway, two RFOs interact to form higher-order RFOs members^11^. Galactinol is synthesized from UDP-D-galactose and myo-inositol by galactinol synthase (GolS; EC 2.4.1.123), which catalyzes the first committed step in the RFOs biosynthetic pathway in plants^12^,^13^,^14^. Thus, GolS is a key regulator in RFOs synthesis and a restriction enzyme for RFOs accumulation^9^,^15^,^16^. Previous studies have shown that GolS was important in the physiology of plant stress resistance, photosynthate translocation and seed physiology. *A. thaliana AtGolS1* and *AtGolS2* were up-regulated by drought and salt stresses, whereas *AtGolS3* was up-regulated by cold stress^6^. *Coffea arabica* L. greatly accumulated *GolS* under drought and salt stresses^17^. *Pisum sativum* L. *PsGolS* increased in roots and epicotyls of seedlings after dehydration, and its level in epicotyls was obviously higher than in roots during dehydration^18^. *AtGolS2* is involved in processes related to seed longevity, but *AtGolS1* does not exert similar action^19^. Some GolS genes are involved in basic metabolic activities, such as carbon storage and the seasonal mobilization of carbohydrates^20^,^21^,^22^. After seed development, seeds accumulate an abundance of RFOs, and GolS activity gradually increases before stachyose rises, reaching its peak after flowering and decreasing to a plateau in dry seeds^13^,^23^,^24^,^25^. Fleshy fruits such as melons actively synthesize raffinose and stachyose, but the contents of both sugars are very low, thereby suggesting that RFOs are rapidly catabolized^26^,^27^. During heat stress, although the *VvGolS1* expression in *Vitis vinifera* increased, galactinol derivatives were not detected upon heat stress, and raffinose and stachyose were merely generated after heat stress^28^. Additionally, plant growth was impaired in GolS-silenced transgenic plants^20^, and GolS activities were related to different isozymes with varying GolS functions^6^,^9^,^17^.

The genus *Ammopiptanthus* (Leguminosae) comprises only two relict species: *A. mongolicus* and *A. nanus* endemically distributed in the South Gobi desert and the desert in Central Asia, respectively. *A. nanus* is distributed only in a narrow altitudinal strip between 1,800 and 2,800 m in the Kezilesu Kirgiz Autonomous Prefecture of Xinjiang and the Soviet Union. In these regions, the temperature varies dramatically from −30 °C to 47.6 °C; at the same time, the climate is extremely arid because annual precipitation is lower than 20 mm, whereas annual evaporation is higher than 2,000 mm^29^,^30^.

To understand the mechanism of abiotic stress tolerance at the molecular level, we acquired a GolS homologous sequence from a cold-induced SSH library derived from *A. nanus* seedlings. The gene was named *AnGolS1*, and its full-length cDNA was cloned. In this paper, it was demonstrated that AnGolS1 possesses the ability to synthesize galactinol in *vitro* and might be an early-responsive gene based on the results of a substrate reaction test. QRT-PCR was used to analyze gene transcript accumulation under different stresses, and the results indicated that *AnGolS1* expression can be responded to cold, salts and drought stresses, significantly up-regulated by low-temperature stress. In addition, *in situ* hybridization (ISH) was utilized to explore *AnGolS1* expression levels in the cells of different tissues under low-temperature stress. Such efforts will help us better understand the mechanism of abiotic stress tolerance, so as to develop and utilize *A.nanus* and to enhance crops toleration.

## Results

### Cloning and Characterization of the Full-length *AnGolS1* cDNA

An *AnGolS1* fragment was obtained from a cold-induced SSH library derived from *A. nanus* seedlings, and then the 5′-end and 3′-end sequences were amplified by 5′- and 3′-RACE, respectively. The full-length cDNA of *AnGolS1* (GenBank Accession No.: GU942748), which had a length of 1,244 bp, was spliced and flanked by stretches of 98 and 159 bp at the 5′- and 3′-untranslated regions, respectively. The cDNA encoded a polypeptide of 328 amino acids with a predicted molecular weight of approximately 37.576 kD.

**Figure 1:**
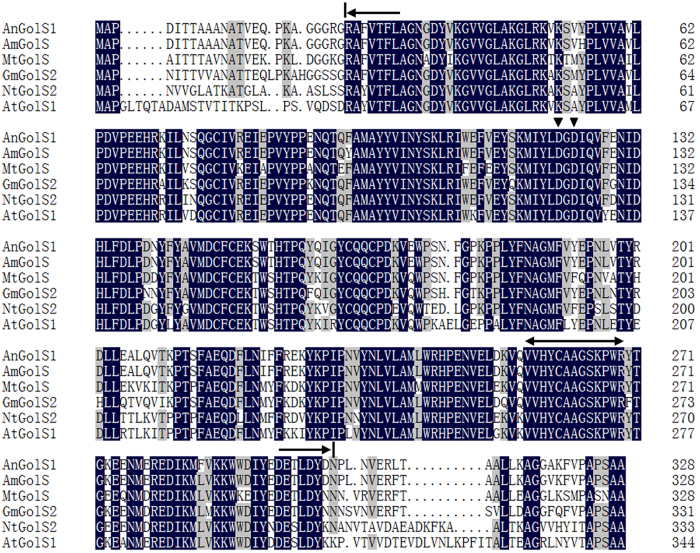
Alignment of the *AnGolS1* amino acid sequence with *AmGolS* (ABF66656.1), *GmGolS2* (XP_003521658.1), *MtGolS* (KEH24464.1), *NtGolS2* (AHM22934.1), and *AtGolS1* (NP_182240.1). The starting and ending sites of the conserved domain are marked with a one-headed arrow. The substrate binding site is marked with a double-headed arrow. The manganese binding site is marked with a triangle.

*AnGolS1* shares a high degree of homology with a variety of GolS proteins from *A. mongolicus (AmGolS*, 98%), *Glycine max (GmGolS1*, 85.80%), *Medicago truncatula (MtGolS*, 82.32%), *Nicotiana tabacum (NtGolS2*, 76.35%), and *A. thaliana (AtGolS1*, 73.26%) (Fig. 1). These homologous proteins, ranged between 328 and 344 amino acids, have seven gaps, six in the region of residues 3 to 35 in the N- and C-termini. Thus, high diversity was found in the first and last 35 positions of the N-termini and C-termini, respectively. In addition, *AnGolS1* includes a substrate binding site and a manganese binding site (Fig. 1), a Pfam domain from amino acids 28 to 272, and a region of low compositional complexity between amino acids 311 and 320.

### Phylogenetic Analysis of *AnGolS1*

**Figure 2:**
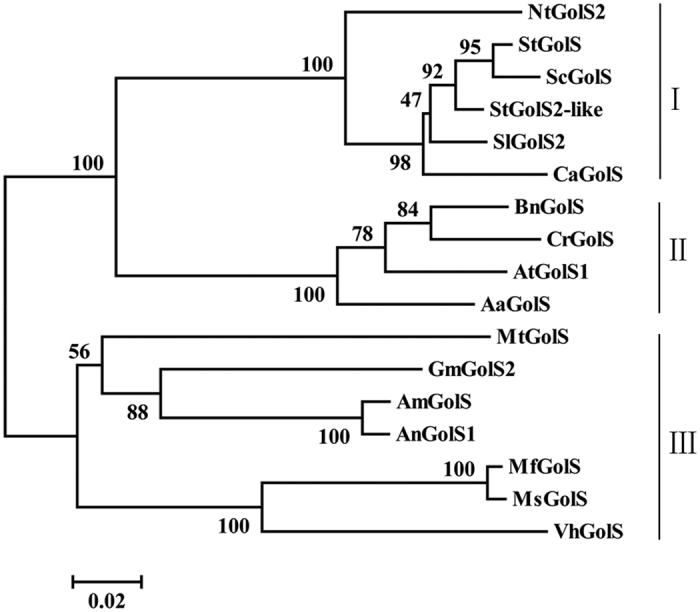
Phylogenetic tree of AnGolS1 and other plant GolS sequences. Evolutionary relationships were inferred using the neighbor-joining method. The bootstrap consensus tree inferred from 1,000 replicates is taken to represent the evolutionary history of the taxa analyzed. The bars represent evolutionary distance. The GolS proteins used in the phylogenetic tree analysis are from the plants *Ammopiptanthus nanus* (AnGolS1, ADF43063.1), *Ammopiptanthus mongolicus* (AmGolS, ABF66656.1), *Glycine max* (GmGolS2, XP_003521658.1), *Medicago truncatula* (MtGolS, KEH24464.1), *Medicago sativa* (MsGolS, AAM97493.1), *Medicago falcata* (MfGolS, ACM50915.1), *Vicia hirsuta* (VhGolS, AGW51291.1), *Nicotiana tabacum* (NtGolS2, AHM22934.1), *Solanum tuberosum* (StGolS2-like, XP_006340652.1), *Arabis alpina* (AaGolS, KFK37461.1), *Solanum tuberosum* (StGolS, ADW78849.1), *Solanum lycopersicum* (SlGolS2, NP_001234668.1), *Brassica napus* (BnGolS, CDX80084.1), *Solanum commersonii* (ScGolS, ADW78842.1), *Capsicum annuum* (CaGolS, ABQ44212.1), *Arabidopsis thaliana* (AtGolS1, NP_182240.1), and *Capsella rubella* (CrGolS, XP_006302453.1).

A phylogenetic tree was constructed to determine the evolutionary relationships among AnGolS1 and other plant GolS proteins based on the alignment of the GolS proteins (Fig. 2). The available GolS proteins could be classified into three major clades (I, II, and III). AnGolS1 grouped with AmGolS, MtGolS, MsGolS, MfGolS, GmGolS2, and VhGolS in clade III. These species in clade III belong to Leguminosae, nevertheless, clades I and II belong to Solanaceae and Brassicaceae, respectively.

### Induced expression and functional characterization of *AnGolS1* in *E. coli*

**Figure 3:**
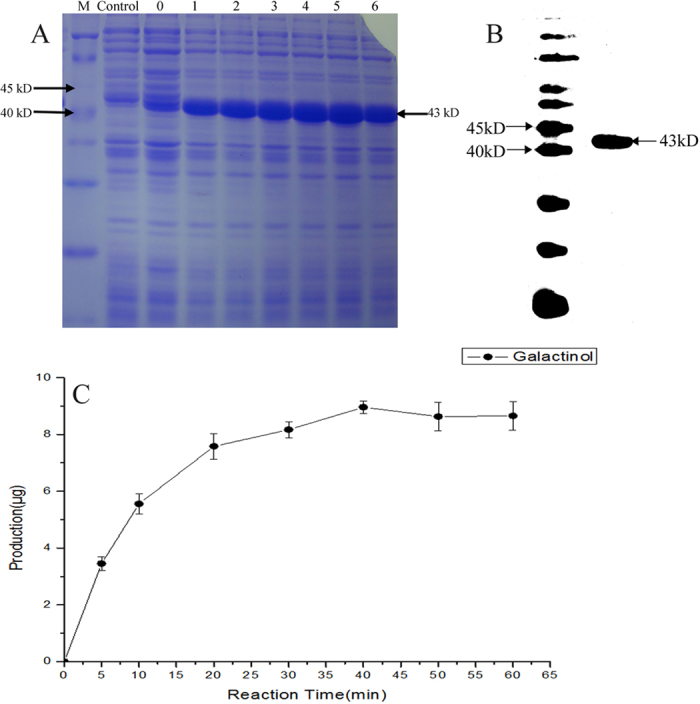
The induction, extraction and analysis of fusion protein enzyme activity. (**A**) Extraction of the induced total proteins. M, protein marker. Control, empty vector (*pET-30a*+) cells were treated with IPTG 6 h. Label 0, *pET-30a-AnGolS1* cells cultured for 6 h under normal conditions. Labels 1 to 6, *pET-30a-AnGolS1* cells treated with IPTG for 1 to 6 h. (**B**) Detection of the purified fusion protein as a band of approximately 43 kD, between 40 kD to 45 kD. (**C**) Changes in the galactinol curve during the substrate reaction test. The horizontal and longitudinal coordinates represent the reaction time and the generation of galactinol, respectively.

A specific 43-kD fusion protein was expressed in BL21 cells which transformed with *pET-30a-AnGolS1* and were induced with isopropyl β-D-1-thiogalactopyranoside (IPTG). The results suggested that the best time point for induction was 5 h, at which time the expressed fusion protein was at maximum levels (Fig. 3A). Thus, we chose to extract fusion proteins from 5 h induced cells (100 mL).

The extracted fusion proteins were used to purify His-fusion proteins, and then the His-fusion proteins were recovered and concentrated. The concentrated proteins had a single vivid 43-kD band but did not exhibit other bands (Fig. 3B). The recovered fusion protein was used in an enzyme activity assay and we found that the reaction product galactinol was rapidly produced after reacting for 5 min at 30 °C, continuously increased at a steady rate for 40 min and then remained at a fairly constant level from 40 min to 60 min (Fig. 3C). These results demonstrated that the fusion protein possessed galactinol synthase activity *in vitro*, and its enzyme activity was very robust.

### Expression analysis of *AnGolS1* in *A. nanus*

**Figure 4:**
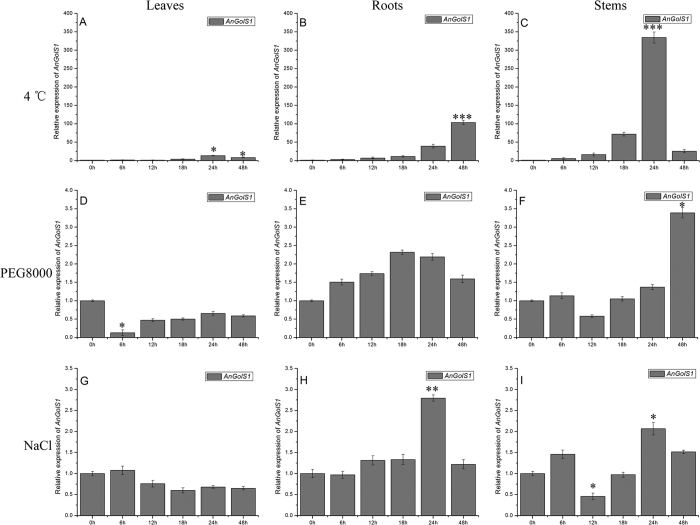
*AnGolS1* expression profiles under different stress treatments. Relative expression in roots, stems and leaves under 4 °C low-temperature treatment (**A**–**C**), under 20% PEG8000 treatment (**D**–**F**) and under 0.25 M NaCl treatment (**G**–**I**). The data were presented as the mean ± SE of three independent experiments. Different letters above the columns indicate significant differences (*P < 0.05, **P < 0.01, ***P < 0.001) according to Duncan’s multiple range test.

qRT-PCR was performed to analyze *AnGolS1* transcript accumulation in *A. nanus* seedlings in different organs under different stresses. Under 4 °C treatment, *AnGolS1* expression in stems and leaves continually increased from 0 h to 24 h and then reduced from 24 h to 48 h, increased respectively 334.5- and 13.4-fold at 24 h compared with controls (Fig. 4A,C), however, that in roots continuously increased throughout the entire duration of treatment and raised 103.6-fold at 48 h (Fig. 4B). Under 20% PEG8000 treatment, the gene expression in stems increased at 4 h and that in leaves significantly decreased 0.13-fold at 6 h compared with controls, but that in roots did not notablely change throughout the entire duration of treatment (Fig. 4D,F). Under 0.25 M NaCl treatment, *AnGolS1* expression increased in roots and stems at 24 h, reduced in stems at 12 h compared with controls (Fig. 4H,I), however, that in leaves did not evidently change (Fig. 4G). Thus, it was suggested that *AnGolS1* was responded to cold, salt and drought stresses, up-regulated by low temperature in all tissues and played a less prominent part in salt and drought stresses compared with low-temperature stress.

### Tissue distribution of *AnGolS1*

**Figure 5:**
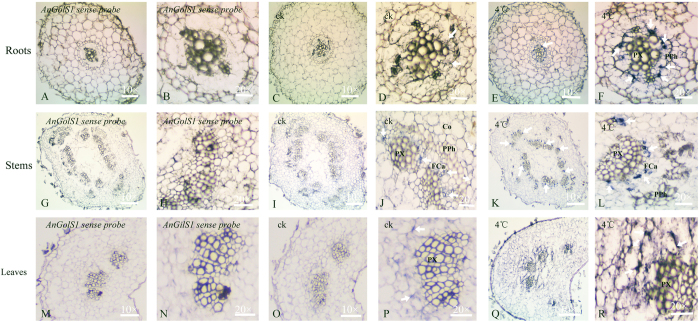
*In situ* hybridization with *AnGolS1* sense and antisense probes of *A. nanus* seedling roots, stems and leaves tissues. Roots, stems and leaves were hybridized with sense probe GST7 (**A**,**B**,**G**,**H**,**M**,**N**) and antisense probe GSSP6 (**C**,**D**,**I**,**J**,**O**,**P**), respectively. Roots, stems and leaves were hybridized with antisense probe GSSP6 after 4 treatment for 24 °C hours (**E**,**F**,**K**,**L**,**Q**,**R**). The white arrows indicated the hybridization signal. PX, primary xylem; PPh, primary phloem; Fca, fascicular cambium; Co, cortex.

**Figure 6:**
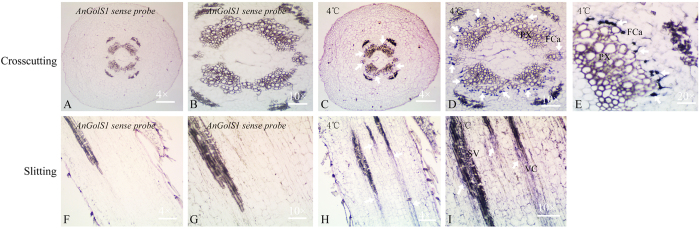
*In situ* hybridization with *AnGolS1* sense and antisense probes of *A. nanus* seedling hypocotyls. The crosscutting hypocotyls were hybridized with sense probe GST7 (**A**,**B**) and antisense probe GSSP6 after cold treatment (**C**–**E**), the slitting hypocotyls were hybridized with GST7 (**F**,**G**), and GSSP6 after cold treatment (**H**,**I**). The white arrows indicated the hybridization signal. VC, vascular bundle; SV, spiral vessel; Fca, fascicular cambium; PX, primary xylem.

*In situ* hybridization was performed on different tissues of *A. nanus*. Two probes (named GST7, sense, and GSSP6, antisense) were used for hybridization (Table S3). The hybridization signal of the antisense probe GSSP6 was intense in the cold treatment tissues and lower in the normal tissues (Fig. 5C,D,I,J,O,P). At low temperature, the signal appeared near the primary phloem and was more noticeable compared with normal roots (Fig. 5E,F). Moreover, a hybridization signal in stems was clearly observed in the fascicular cambium between the phloem and xylem and was many times stronger than in normal stems (Fig. 5K,L). A stronger hybridization signal was observed in fascicular cambium hypocotyls at low temperature (Fig. 6). In addition, a strong hybridization signal still appeared in leaves under low temperature (Fig. 5Q,R). Meanwhile, the negative-control hybridizations with the sense-strand probe GST7 did not result in signal detection in all tissues (Fig. 5A,B,G,H,M,M). These results were consistent with the *AnGolS1* transcript expression results and further confirmed that cold stress significantly regulated *AnGolS1* accumulation.

## Discussion

*A. nanus* has evolved a very strong ability for cold tolerance and is suitable to study the cryoprotectant mechanisms of woody species. To understand the cryoprotectant mechanisms of *A. nanus*, the full-length *AnGolS1* gene was cloned and its possible function was analyzed from various perspectives.

Different GolS enzymes exhibit different penta-peptide characteristics in their C termini, such as AnGolS1, AmGolS, NtGolS2 and AtGolS1 express the characteristics of APSAA, whereas MtGolS shows ASNAA characteristics (Fig. 1). In addition, XvGolS, ArGolS1 and BnGolS also emerge APSAA characteristics^31^, BhGolS1 demonstrates PPTA characteristics^32^ and ZmGolS37 only has a proline residue in its C terminus, but other ZmGolS genes demonstrate APSAA characteristics^33^. The differences in the termini are likely connected with GolS function. Furthermore, the AnGolS1 protein does not possess a signal peptide site, which suggested that this protein is not a secreted protein.

Galactinol, which is synthesized by GolS, is an essential substrate for the synthesis of RFOs. Thus, GolS activity limits RFO accumulation. In *AtGolS1* mutant plants, galactinol and RFOs did not accumulate under heat-induced stress^34^, and the double mutants (*AtGolS1* T-DNA insertion mutant on an *AtGolS2* background) did not tolerate drought tolerance^35^. Previous studies demonstrated that RFOs possess a cryoprotectant role for photosystem II (PS II), and the level of galactinol in the *A. thaliana* raffinose synthase (RS14) mutant was higher than that in the wild-type plants during cold treatment^36^. The dehydration-responsive element binding proteins (DREB1A/CBF3) and C-repeat binding factor (CBF1) transgenic plants accumulated large amounts of galactinol and RFOs, and plant cold tolerance was obviously improved^37^,^38^,^39^,^40^. Fundamentally, the increased expression of *AtGolS3* directly caused increased RFO levels in DREB1A transgenic plants^37^. In qRT-PCR experiments, we found that *AnGolS1* was significantly up-regulated in all organs by low-temperature stress, and its expression in stems was higher 334.5-fold than that in controls (Fig. 4C). Thus, two assumptions were made to predict the possible function of *AnGolS1* at the tissue level. First, galactinol was rapidly synthesized in stems by *AnGolS1*, and then the majority was utilized to synthesize RFOs by raffinose synthase under low-temperature stress. Second, only a small portion of synthesized galactinol was utilized to synthesize RFOs; most galactinol directly acted to protect plants independently as it was found that RFOs and galactinol could protect plant cells from oxidative damage under low temperature^41^. Besides, both the CsGolS1 overexpressing and galactinol-treated wild-type tobacco plants enhanced the resistance against pathogen infection and drought and high salinity stresses^42^. These results indicated that galactinol has a protecting function in biotic and abiotic stresses in plants. In addition, its expression in roots and leaves increased under low temperature, suggesting that this protection mechanism was also present in roots and leaves (Fig. 4A,B). Expression in roots and stems at 2 h increased under salt stress, but the varieties were smaller compared with the same tissues under low-temperature stress. Furthermore, similar variations were evident in stems at 48 h under PEG8000 stress.

In different plants, the *GolS* genes demonstrate differential tissue-specific expression. For example, *CaGolS1* had relatively higher transcript abundance in coffee leaves than *CaGolS2* and *CaGolS3*, but *CaGolS2* was not present or low express in almost all tissues and *CaGolS3* had higher abundance in flowers and roots than in leaves^17^. *GhGolS1* in anthers was considerably higher than in leaves^43^. *ZmGolS2* was regulated by drought stress, but *ZmGolS3* was only present in mature seeds as a stored message^44^. Although the AnGolS1 protein demonstrates high similarity to AmGolS, MfGolS1, MsGolS, MtGolS, GmGolS2 and VhGolS, the functions of these proteins have large differences. *MfGolS1* was up-regulated after cold treatment in senescent leaves, mature leaves and lateral roots but was not expressed in the petiole, stems and axial roots, and it was weakly induced by dehydration and salt stresses^45^. Moreover, *MsGolS* was also induced by cold stress^45^. Furthermore, the *AmGolS* expression began to increase at 12 h under a temperature of 4 °C, thus indicating that it is a late-responsive gene similar to *AtGolS3*^6^, and it was regulated by drought, high salinity and ABA stresses^46^. However, although *AnGolS1* can been regulated by PEG8000, salt and low-temperature, *AnGolS1* is mainly up-regulated by low temperature, and its expression in stems at 6 h was increased 5.6-fold compared with controls. In addition, galactinol increased to 2 μg after reacting for 5 min during a substrate reaction test. Thus, we believe that *AnGolS1* is likely to be an early-responsive gene. Besides, its expressions in leaves were depressed under salt or PEG8000 stresses.

It has been verified that *Ajuga reptans* plants contain two RFOs pools: one is an RFO storage pool in the mesophyll, and the other is an RFO transport pool in the companion cells of the phloem^47^. In warm- and cold-grown leaves of *Ajuga reptans*, the hybridization signals of *ArGolS1* and *ArGolS2* were observed in the mesophyll and phloem-associated intermediary cells, respectively, in ISH studies; thus, *ArGols1* and *ArGolS2* were speculated to associate with the synthesis of storage and transport RFOs, respectively^48^. In this paper, we observed that the hybridization signal in stems was very strongest in all tissues after low-temperature treatment and that the signal was mainly focused in the fascicular cambium surrounding the primary xylem. This appearance was observed in the fascicular cambium in crosscut hypocotyls and near the vascular bundle following slitting after low-temperature treatment. At the same time, we also found that hybridization signals clearly emerged in roots and leaves surrounding the primary xylem, but their signal strength was weaker than that in stems. Thus, we speculated that *AnGolS1* is likely to be first synthesized in the fascicular cambium in stems and then synthesized galactinol was transported in proximity to other cells, such as the cortex, phloem and xylem, under low-temperature stress. More studies must be carried out to explore the function of the fascicular cambium cells in the cold tolerance of *A. nanus* and to determine how these cells regulate *AnGolS1* gene expression.

In conclusion, we believe that *AnGolS1* might aid *A. nanus* in resisting the harmful effects of low-temperature stress, and fascicular cambium cells could be involved in *AnGolS1* mRNA transcription and then galactinol transportation and coordination under low-temperature stress. These hypotheses not only help us to better understand the important role that *AnGolS1* plays in phloem loading, but will also further guide us to explore the metabolism regulatory mechanisms of low-temperature stress in *A. nanus* and enhance crop resistance via production practices employing transgenic technology.

## Materials and Methods

### Plant Materials

*A. nanus* seeds were collected from Wuqia County in Xinjiang Provence in China and cultivated in a sandy substrate with a relative humidity of approximately 60%. The seedlings with 6 to 8 leaves (grown for approximately 6–8 weeks) were subjected to 4 °C low-temperature, 0.25 M NaCl, or 20% PEG 8000 (instead of control watering) stresses, and each stress was, respectively assayed after 0, 6, 12, 18, 24 and 48 h of treatment. The roots, stems and leaves of these seedlings at different treatment times were gathered and utilized as samples for qRT-PCR. In addition, the roots, stems and leaves of seedlings at 24 h after low-temperature stress were utilized for *in situ* hybridization.

### Primers

The primers used in this study are shown in Table S3.

### RNA Extraction and cDNA Synthesis

Total RNA from roots, stems and leaves was individually extracted using the TaKaRa MiniBEST Plant Universal RNA Extraction Kit (Takara, Japan). First-strand cDNA was synthesized using Superscript III Reverse Transcriptase (Invitrogen, America).

### 5′-RACE and 3′-RACE

Based on the GolS segment sequence, gene-specific primers were designed and synthesized (Table S3). The 5′- and 3′-end sequences were obtained using the 5′ and 3′ RACE System for Rapid Amplification of cDNA Ends (Invitrogen). The inner 5′ and 3′ RACE PCR products were resolved on agarose gels, and the appropriate bands were excised and then purified and cloned into the pMD18-T vector for sequencing.

### Full-length cDNA Amplification of *AnGolS1*

The full-length cDNA of *AnGolS1* was deduced by sequence comparison and alignment with the 5′-RACE and 3′-RACE sequences using DNAMAN software. The AnGS F and AnGS R primers were designed based on the deduced cDNA to obtain the full-length sequence (Fig. S2A). The purified PCR product was cloned into the pMD18-T vector for sequencing as described above.

### *AnGolS1* fusion protein expression

*AnGolS1* was expressed in *Escherichia coli* as a His-fusion protein using the plasmid vector *pET-30a* (+). The coding region of AnGolS1 was amplified with two primers (forward, AnGS F; reverse, AnGS R) that contained BamHI and HindIII restriction sites, respectively. After digestion with HindIII/BamHI, the fragment was inserted into *pET-30a* (+) using the same restriction sites and the recombinant vector was designated *pET-30a-AnGolS1* (Fig. S2B). Empty *pET-30a* (+) and *pET-30a-AnGolS1* were transformed into *E. coli* BL21 (DE3) cells.

*E. coli* cells transformed with *pET-30a-AnGolS1* were treated with 1 mmol.L^−1^ IPTG and cultured for different amounts of time. Finally, 1 mL of treated cells were harvested, boiled and analyzed by Coomassie blue staining using 15% SDS–polyacrylamide gels.

### Enzyme activity assay for fusion proteins

Total *E. coli* proteins were extracted from treated cells (100 mL) and purified with His-fusion proteins using Ni-NTA His-Bind Resin (Merck), and then enzyme activities were recovered using phosphate buffered saline (PBS). GolS activity was determined according to the method described by Peterbauer^49^. The final reaction volume (30 μL) for the GolS activity assay contained 10 μL of the recovered enzyme extract, 5 mM MnCl_2_, 5 mM UDP-galactose (Sigma), and 20 mM myo-inositol. To better assay the resulting quantity of galactinol, we designed a reaction time gradient, and after incubation at 30 °C, the reaction was terminated by adding 70 μL of 80% ethanol and boiling the mixture for 5 min. The extracted reaction products (10 μL) were analyzed by High Performance Liquid Chromatography (HPLC) with pulsed amperometric detection utilizing a Carbopac PA10 column (Dionex, Vienna)^50^. Control reactions contained PBS buffer instead of substrates (Fig. S3; Tables S1 and S2).

### Bioinformatics Analyses

DNASTAR software was used to translate the open reading frame (ORF) and to calculate the molecular weight of the deduced protein. BLAST was performed on the NCBI server (http://www.ncbi.nlm.nih.gov/BLAST). The SignalP 4.1 Server was used to predict protein signal peptides (http://www.cbs.dtu.dk/services/SignalP/). Protein conserved domains were predicted on the NCBI server (http://www.ncbi.nlm.nih.gov/Structure/cdd/wrpsb.cgi) and the SMART server (http://smart.embl-heidelberg.de/). Multiple sequence alignments and phylogenetic analysis were performed by using the ClustalW^51^ and GeneDoc^52^ programs. A phylogenetic tree was constructed using MEGA 4.0^53^ from ClustalW alignments and the neighbor-joining method^54^ was used to construct a tree. Structural analysis of the deduced proteins was accomplished using SWISS-MODEL^55^,^56^ on the website (http://www.expasy.org), and WebLab ViewerLite 4.0 was used to display the 3D model (Fig. S1).

### *AnGolS1* expression in *A. nanus*

qRT-PCR was used to analyze *AnGolS1* transcript accumulation in different tissues of *A. nanus* seedlings in a greenhouse under different stresses grown. Total RNA was isolated from leaves, stems, and roots by using the TaKaRa MiniBEST Universal RNA Extraction Kit (Takara, Japan), and first-strand cDNA was synthesized as mentioned above. This cDNA was used as a template in qRT-PCR with the primers AnGolS1 RT-F and AnGolS1 RT-R, which are specific to the *AnGolS1* coding sequence. Actin-F and Actin-R primers were used to amplify the housekeeping gene Actin as an internal control. The qRT-PCR reactions were performed in triplicate for each sample using Real Master Mix (SYBR Green) (Tiangen, China) on a real-time PCR system (ABI 7500, USA) under the following conditions: 95 °C for 60 s, 40 cycles of 95 °C for 15 s, 60 °C for 30 s, and 68 °C for 60 s, 1 cycle of 95 °C for 15 s and 60 °C for 1 min, and 95 °C for 15 s. The specificity of each primer pair was validated by a dissociation curve. The experiment was repeated at least three times, and gene expression levels were calculated based on the method described by Pfaffl^57^. Significant differences in the data were analyzed according to Duncan’s multiple range test.

### Synthesis and DIG-labeled probe

The primers GS-HF and GS-HR were used to synthesize the probe. The probe was labeled with digoxigenin-UTP via *in vitro* transcription with SP6 and T7 RNA polymerase following the manufacturer’s protocol (DIG RNA Labeling Kit (SP6/T7), Roche) (Fig. S4A,B).

### Collection and fixation of *A. nanus* specimens

The roots, stems and leaves of seedlings at 24 h after low-temperature treatment were collected individually. After the specimens were fixed in a solution consisting of 2% paraformaldehyde, 2.5% glutaraldehyde, and 0.1 M NaH_2_PO_4_/Na_2_HPO_4_ (pH 7.4) at 4 °C overnight, dehydrated, cleared through an ethanol-to-xylene series and embedded in paraffin, as described by Pirttilä^58^.

### *In Situ* Hybridization (ISH) protocol

The ISH protocol was modified from the recommended method of Abcam (http://www.abcam.com/protocols/ish-*in-situ*-hybridization-protocol). Paraffin-embedded tissue sections at 9 μm were dewaxed with xylene and rehydrated through a series of gradient-diluted ethanol solutions. Next, the slices were successively treated with proteinase K (Roche), acetic acid, gradient-diluted ethanol solutions and were air-dried. After pre-hybridization at 55 °C for 1 h, hybridization was carried out in a solution containing heat-denatured probe by incubating at 55 °C overnight. Post-hybridization was performed through a serially diluted saline-sodium citrate (SSC) gradient, followed by treatment with maleic acid buffer containing Tween 20. The slides were successively blocked with DNA blocking reagent (Roche) and detected with an anti-digoxigenin-AP Fab fragment (Roche) for 1 h at room temperature. The antibody-antigen complexes were subsequently visualized with NBT/BCIP (Roche) at a 1:50 dilution in alkaline buffer at 37 °C overnight. Finally, the slides were rinsed with distilled water for 5 min and air-dried.

## Additional Information

**How to cite this article**: Liu, Y.D. *et al.* Molecular cloning and expression of an encoding galactinol synthase gene (*AnGolS1*) in seedling of *Ammopiptanthus nanus. Sci. Rep.* **6**, 36113; doi: 10.1038/srep36113 (2016).

**Publisher’s note:** Springer Nature remains neutral with regard to jurisdictional claims in published maps and institutional affiliations.

## Supplementary Material

Supplementary Information
