# Supplementary material for: Molecular cloning and expression of an encoding galactinol synthase gene (AnGolS1) in seedling of Ammopiptanthus nanus
Source: Sci Rep. 2016 Oct 27;6:36113. doi: 10.1038/srep36113 (PMC5081558; doi:10.1038/srep36113)
Supplement: Supplementary Information [file srep36113-s1.pdf]

Supplementary Information

Molecular cloning and expression of an encoding galactinol synthase gene (*AnGolS1*) in seedling of *Ammopiptanthus nanus*

YuDong Liu, Li Zhang, LiJing Chen, Hui Ma, YanYe Ruan, Tao Xu, ChuanQiang Xu, Yi He, MingFang Qi

1. Horticulture Department, Shenyang Agricultural University, No. 120 Dongling Road, Shenhe District 110866, PR China

2. Key Laboratory of Protected Horticulture of Ministry of Education, No.120 Dongling Road, Shenhe District 110866, PR China

3. Key Laboratory of Protected Horticulture of Liaoning Province, No. 120 Dongling Road, Shenhe District 110866, PR China

4. Key laboratory of agricultural biotechnology of Liaoning Province, No. 120 Dongling Road, Shenhe District 110866, PR China

5. Collaborative Innovation Center of Protected Vegetable Suround Bohai Gulf Region, No. 120 Dongling Road, Shenhe District 110866, PR China

Horticulture Department, Shenyang Agricultural University, No. 120 Dongling Road, Shenhe District , Shenyang 110866, P R China

Tel No. +86-24-88342256

Fax No. +86-24-88342256

E-mail: qimingfang@126.com

Supplementary Figures

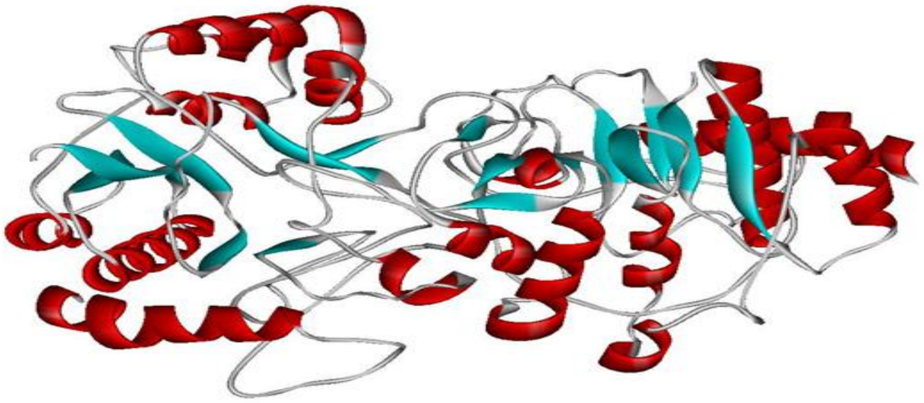

Figure S1. The predicted 3D structure of *AnGolS1*. The 3D structure of *AnGolS1* resembled two connected half ball, the left half ball formed with 9 strands and 8 helices, the right half ball formed with 8 strands and 8 helices**.**

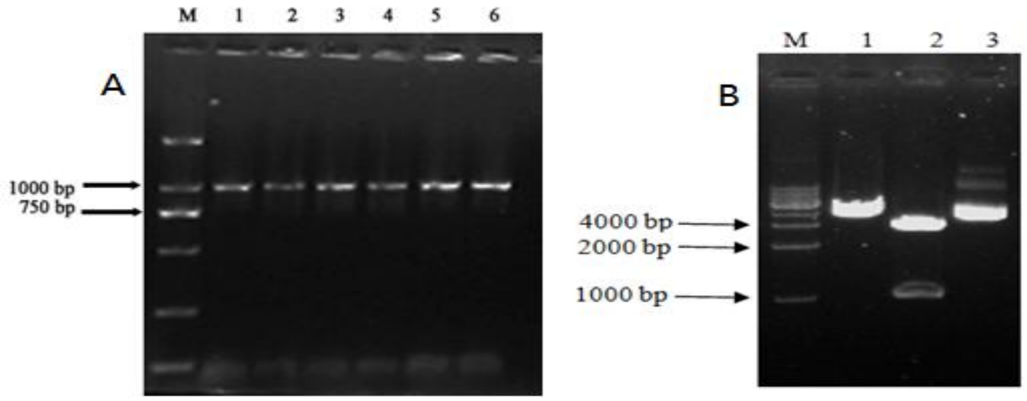

Figure S2. (A) The CDS of *AnGolS1* clone. The PCR programs contained 2 μL of cDNA, 5μL 10 × buffer, 1 mM of primer pair, 0.2 mM of dNTPs, 1.5 mM of $MgSO_4$, 1U of KOD-Plus-Neo DNA polymerase [Toyobo, Japan], and sterile double distilled water to the final volume of 50 μL. M, DL2000 Marker; 1-6 lane, Tm value 55, 56, 57, 58, 59, 60 and 62 ℃, respectively. (B) The identification of recombinant plasmid by double restriction endonuclease digestion. The restriction programs contained 1 μg of recombinant plasmid, 2 μL 10×reaction buffer, 10U of BamHI/HindIII restriction enzyme (Thermo), and sterile double distilled water to the final volume of 20 μL. M, 1kb DNA Ladder; 1 lane, BamHI signle restriction endonuclease digestion; 2 lane, BamHI + HindIII digestion; 3 lane, no digestion.

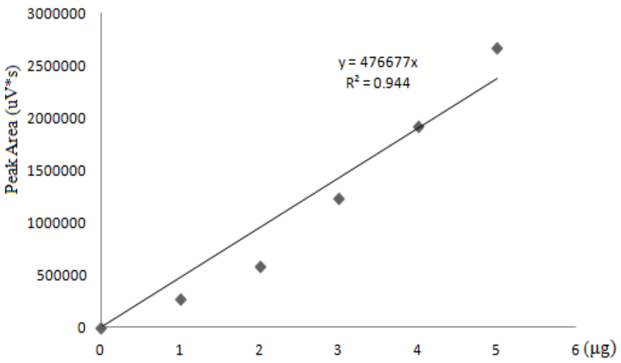

Figure S3. The standard curve of Galactinol. X-axis, quantity (μg); Y-axis, peak area (uV*s).

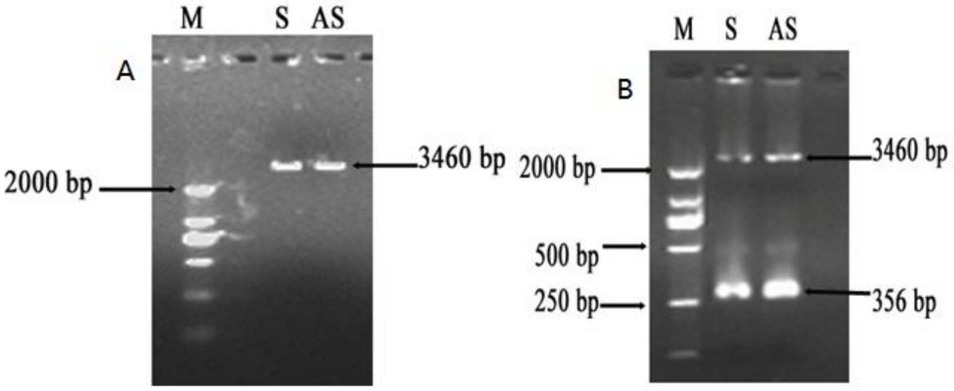

Figure S4. (A) The single restriction endonuclease digestion of ISH *pSPT19* recombinant plasmid. M, DL2000 Marker; S, BamH I digestion sense probe recombinant plasmid; AS, Ecor I digestion antisense probe recombinant plasmid. (B) The identification of ISH probes. M, DL2000 Marker; S, Sense probe (T7 RNA polymerase)；AS, Antisense probe (SP6 RNA polymerase). The 3460 bp band represent linearized plasmid DNA; the 356 bp band represent synthesized single DIG-labeled RNA probes.

Supplementary Tables

Table S1. The analysis data of standard curve.

Sample concentration (mg/mL)	Sample Volume (μL)	Peak Area (uV*s)	Quantity (μg)
0.2	5.0	271501	1
0.4	5.0	587562	2
0.6	5.0	1233202	3
0.8	5.0	1927052	4
1.0	5.0	2672562	5

Table S2. The analysis data of reaction products in enzyme activity assay.

Total Reaction Volume(μL)	Reaction Time (min)	Sample Volume (μL)	Peak Area (uV*s)	Quantity (μg)
30	5	5.0	164412	2.069477
30	10	5.0	286537	3.606681
30	20	5.0	369635	4.652647
30	30	5.0	389291	4.90006
30	40	5.0	426947	5.374042
30	50	5.0	411294	5.177015
30	60	5.0	412244	5.188973

Table S3. The sequences of primers during this study.

Primer name	Sequence(5′ -′ 3)	Description
AAP	GGCCACGCGTCGACTAGTACGGGIIGGGIIGGGIIG	5'RACE the first PCR adapter primer
AUAP	GGCCACGCGTCGACTAGTAC	5'RACE the second PCR adapter primer
UAP	CUACUACUACUAGGCCACGCGTCGACTAGTAC	5'RACE the third PCR adapter primer
AP	GGCCACGCGTCGACTAGTACTTTTTTTTTTTTTTTTT	Universal adapter primer, outer
AP1	GGCCACGCGTCGACTAGTAC	Universal primer, nested
3-GS1	AGCCTAATCTCGTCACTTACCGT	3′-RACE forward primer, outer
3-GS2	CAGGGAAAAATACAAGCCAATAC	3′-RACE forward primer, inner
5-GS	TTGAAATAGAGAGGAGGCTTGG	5'RACE Reverse Transcript gene primer
5-GS1	CCAATCTGATACTGAGGGGTGTG	5'RACE the first PCR gene primer
5-GS2	TGAGGGGTGTGGGTCCAACTT	5'RACE the second PCR gene primer
5-GS3	TCACAGAAACAATCCATAACCGC	5'RACE the third PCR gene primer
AnGS F	AAGGATCC ATGGCACCTGATATCACCACCG	Full length cDNA sequence primer, forward (BamHI)
AnGS R	GGAAGCTTTTAGGCAGCGGATGGGGC	Full length cDNA sequence primer, reverse (HindIII)
AnGS RT-F	ACGAATTTGGGAGTTTGTGGAG	Real-time PCR primer, forward

AnGS RT-R	AATAGAGAGGAGGCTTGGGACC	Real-time PCR primer, reverse
Actin F	ACATTGTCTTGAGTGGTGGTTC	Standard control primer, forward
Actin R	TACTTCCTCTCTGGTGGTGCTA	Standard control primer, reverse
AnGS SF	AA*GGATCC*GCTAAAGGACTTAGGAAGGTGAA	Sense probe GST7 PCR primer, forward (EcoRI)
AnGS SR	GT*GAATTC*CCAATCTGATACTGAGGGGTGTG	Sense probe GST7 PCR primer, reverse (BamHI)
AnGS ASF	GT*GAATTC*GCTAAAGGACTTAGGAAGGTGAA	Antisense probe GSSP6 PCR primer, forward (BamHI)
AnGS ASR	AA*GGATCC*CCAATCTGATACTGAGGGGTGTG	Antisense probe GSSP6 PCR primer, reverse (EcoRI)
